# Prevalence, Awareness, Treatment, and Control of Hypertension and Its Risk Factors in (Central) Vietnam

**DOI:** 10.1155/2018/6326984

**Published:** 2018-05-16

**Authors:** Ho Anh Hien, Nguyen Minh Tam, Vo Tam, Anselme Derese, Dirk Devroey

**Affiliations:** ^1^Department of Family Medicine, Hue University of Medicine and Pharmacy, Hue University, Hue, Vietnam; ^2^Department of Family Medicine and Chronic Care, Faculty of Medicine and Pharmacy, Vrije Universiteit Brussel, Brussels, Belgium; ^3^Department of Internal Medicine, Hue University of Medicine and Pharmacy, Hue University, Hue, Vietnam; ^4^Department of Family Medicine and Primary Health Care, Faculty of Medicine and Health Sciences, Ghent University, Ghent, Belgium

## Abstract

**Introduction:**

The objective of this study is to describe the prevalence, awareness, treatment, and control of hypertension and its associated risk factors in (Central) Vietnam.

**Methods:**

In this cross-sectional study, a multistage sampling was used to select 969 participants from the general population aged from 40 to 69 years. The cardiovascular risk factors were collected throughout the interviews with a standardized questionnaire. Multivariate logistic regression analysis was conducted to test the relationship between the prevalence, awareness, treatment, and control of hypertension and the prevalence of risk factors.

**Results:**

The prevalence of hypertension was 44.8%. It was higher in men than in women (51.3% versus 39.7%, *p* < 0.001). In total 67.3% (74.5% in women, 60.1% in men; *p* = 0.001) of the participants were aware of their hypertension, 33.2% (37.5% in women, 28.9% in men; *p* = 0.01) of the participants were treated, and 12.2% (16.7% in women, 7.8% in men; *p* < 0.001) of the hypertensive participants' hypertension was controlled. Age, gender, place of residence, body mass index, and diabetes were found to be independent risk factors for hypertension.

**Conclusion:**

The prevalence of hypertension in Vietnam is high, and the proportion of treated and controlled patients is rather low.

## 1. Introduction

Hypertension is one of the most important modifiable risk factors for cardiovascular diseases (CVD) worldwide. The complications of hypertension account for 9.4 million deaths worldwide every year [1]. Hypertension is responsible for at least 45% of the deaths from CVD and 51% of the deaths from stroke [2]. CVD accounted for more than 17.3 million deaths in 2013 which represented 31% of all global deaths and made it the world's leading cause of death and disability [1]. Especially in low- and middle-income countries, CVD accounts for around 70% of deaths [1]. In Vietnam, the prevalence of hypertension has rapidly increased over the last two decades from 16.9% over the period of 2001-2002 to 25.1% in 2008 [3]. In 2009, CVD was estimated to account for 33% of all deaths [4] and stroke was found to be the leading cause of death among them [5, 6]. At that time, the percentages of treatment and control of hypertension were rather low (29,6% and 10,7%, resp.) [3].

In other developing countries, the mean prevalence, awareness, treatment, and control of hypertension were 32.2, 40.6, 29.2, and 9.8 percent among men and 30.5, 52.7, 40.5, and 16.2 percent among women, respectively. In developed countries, these percentages in men were 40.8, 49.2, 29.1, and 10.8 percent, and in women they were 33.0, 61.7, 40.6, and 17.3 percent, respectively [7]. A successful hypertension detection and treatment program in Canada resulted in a large increase of the diagnosis of hypertension from 57% in 1992 to 84% in 2013 and a reduction in the national rates of death and hospitalization due to CVD. Several cross-sectional studies had been implemented to better understand the approach of hypertension in this country, resulting in a better prevention and treatment [8].

The impact of hypertension and CVD is influenced by a wide variety of risk factors such as tobacco use, excessive alcohol consumption, unhealthy diet, physical inactivity, overweight and obesity, elevated blood glucose, and abnormal blood lipids. The combination of a reduction of risk factors in the general population, primary prevention in high-risk groups, and intensive treatment in secondary prevention was claimed to be the best strategy to reduce CVD premature mortality [9]. Research from several countries has consistently shown that the treatment of risk factors such as hypertension has a higher impact on CVD than the treatment of established CVD [10, 11].

The prevalence of hypertension has significantly increased due to the rapid economic development, population aging, urbanization, and changes in dietary habits and lifestyle in Vietnam. From 2005 to 2011, few studies have attempted to explore the awareness, treatment, and control of hypertension in Vietnam [3, 12–14]. The increase in the prevalence of hypertension requires an improved approach of hypertension management; however, this kind of study was not conducted over the past 10 years in (Central) Vietnam or at least in Thua Thien Hue Province. There remained also little information on the influence of sociodemographic and risk factors on the awareness and control of hypertension. Therefore, the objectives of this study were to describe the prevalence, awareness, treatment, and control of hypertension and its risk factors in Thua Thien Hue Province in Central Vietnam. The findings of this study are expected to contribute to primary prevention and intervention strategies in the treatment of hypertension in Central Vietnam.

## 2. Methods

### 2.1. Study Design and Population

A cross-sectional study was designed to assess the characteristics and risk factors of patients with hypertension in (Central) Vietnam. The present study used data collected in a population-based survey that was carried out among residents of Thua Thien Hue Province in 2015 (population = 1,123,800, census 2013, Statistical Yearbook of Vietnam). Multistaged sampling methods were used to recruit a representative sample of the general population aged from 40 to 69 years. At first, one province, namely, Thua Thien Hue, was purposively selected to register the characteristics of Central Vietnam. Next, we selected one urban district, one low-lying district, and one mountainous district to obtain a sample representing the diversity of this province. Hue City was the only urban district in this province; Phu Loc District and Nam Dong District were selected as low-lying and mountainous districts, respectively. In each district, we randomly selected two communes, yielding a total of 6 selected communes in Thua Thien Hue Province. Finally, from those communes, we used the population list from the local government registers of household updated by 2013. We then selected those aged between 40 and 69 years from the list. After that, 200 persons aged 40 to 69 years were systematically selected from that population list per commune at an interval of every 10th unit. In total, there were 400 people selected in each district, resulting in 1200 selected participants in the whole province.

### 2.2. Data Collection

Twelve hundred randomly selected persons were invited to visit the six local community health centers in the morning on dates indicated in the invitation letters. In each letter, we informed the participants about the time, the site, and other dietary requirements. They were asked not to drink or eat and to bring accurate information about their blood pressure and blood glucose. First, the participants were asked some questions on their demographics and hypertension risk factors such as their current smoking status, alcohol consumption, and physical activity. At the second step, their blood pressure and capillary blood glucose were collected with anthropometric measurement. In total, 983 participants showed up in the local community health centers, resulting in a response rate of 81,9%. Fourteen participants were excluded because of lacking information in the risk factors. The survey teams consisted of medical doctors and medical students from Hue University of Medicine and Pharmacy, Hue University, along with local healthcare providers. All study investigators and staff members had successfully completed a training program that oriented them both on the aims of the study and the specific instruments and methodologies employed. Two trained medical students performed anthropometric measurements. A team of 15 doctors and medical students were in charge of the interviews and physical examinations using a standardized questionnaire based on the World Health Organization (WHO) stepwise approach [15]. The survey was conducted from April to October in 2015.

### 2.3. Study Variables

Demographic data (including age, gender, education, ethnicity, and occupation) were collected. The interviews included questions related to personal and family medical history of hypertension, CVD, smoking, alcohol consumption, physical activity, and fruit and vegetable consumption. Information on the awareness of and treatment for hypertension, body weight, height, waist and hip circumference, blood pressure, and glucose was also obtained. Anthropometric measurements were performed with the participants wearing light clothes without footwear. Body weight was measured to the nearest 0.1 kg using a digital scale (OMROM HN 283, Omron Healthcare, Tokyo, Japan) and height was recorded to the nearest 0.1 cm in the standing position using a Telescopic Measuring Rod (MZ10023-3, ADE, Germany). Waist circumference was recorded to the nearest 0.1 cm, using a tape measure (Hoechstmass tape 84.203, Sulzbach, Germany), directly over the skin, at the level of the midpoint between the inferior margin of the last rib and the iliac crest in the mid-axillary line. Hip circumference was also recorded to nearest 0.1 cm, using a tape measure directly over the skin, around the point with the maximum circumference over the buttocks.

Blood pressure was measured on at least two occasions with a minimum interval of 15 minutes in resting and sitting position, using automatic sphygmomanometers (OMRON HEM 7322, Omron Healthcare, Tokyo, Japan), with an appropriate sized cuff and a standard protocol. If the difference between the first two measurements was more than 10 mmHg, a third measurement was performed. The average of the last 2 measurements was calculated and used in the analysis. Fasting capillary blood glucose was measured by means of a glucometer (OneTouch Ultra 2, LifeScan, US) using the standard protocol, provided by the manufacturer.

The participants' occupational status was classified into three categories: (1)* experts and government staff*; (2)* manual worker *(farmers and building and production workers); and (3)* other jobs* such as small traders, housekeepers, housewives, and those unemployed. There were three educational levels: (1)* primary school or under* (<6 years of education), (2)* middle or high school* (≥6 years but ≤ 12 years), and (3)* college or university* (graduates of college or university). The vast majority of the respondents belonged to the ethnic majority of Vietnam, namely, “Kinh.” The ethnic minorities included “Ta Oi,” “Van Kieu,” and “Co Tu.”* Urban and rural residence *was identified on an administrative basis for each commune within each province*. Current smokers* were defined as those currently consuming tobacco products (cigarettes, cigars, and pipes) or having quit smoking for less than 12 months. The category* excessive alcohol consumption* included men who took more than 2 standard units per day or 14 standard units per week and women who took more than 1 standard unit of drink per day or 7 standard units of drink per week. Physical activity was categorized by metabolic equivalent tasks per minute per week (MET/min/week).* Physical inactivity (low level of physical activity)* referred to people with fewer than 600 MET/min/week of physical activity, moderate level of physical activity (601–3000 MET/min/week), and high level of physical activity (>3000 MET/min/week). Body mass index (BMI) was calculated as weight (kg) per height squared (m^2^). At the time of our survey, underweight was defined by WHO Regional Office for Western Pacific as BMI < 18.5 kg/m^2^;* normal weight* was defined as BMI from 18.5 to 22.9 kg/m^2^;* overweight* was defined as BMI from 23 to 24.9 kg/m^2^ and* obesity* was defined as BMI ≥ 25 kg/m^2^ [16].* Abdominal obesity* for the Asian population included those whose waist hip ratio was valued ≥0.9 for men or ≥0.8 for women [17].

*Hypertension* was defined as an average systolic blood pressure (SBP) ≥ 140 mmHg and/or average diastolic blood pressure (DBP) ≥ 90 mmHg and/or self-reported previous diagnosis of hypertension by a health professional [18].* Awareness of hypertension* was defined as the self-report or any prior diagnosis of hypertension by a healthcare professional among the participants defined as having hypertension.* Treatment of hypertension* was defined as use of a prescriptive medication for management of the respondent's high blood pressure (BP) for at least 2 weeks at the time of the interview. Treatment was calculated among patients who were fully aware of their hypertension and among the whole hypertensive group (including those who were not aware).* Control of hypertension* was defined as pharmacological treatment of hypertension associated with an average SBP < 140 mmHg and an average DBP < 90 mmHg. The proportion of hypertension control was calculated as the hypertensive patients who were treated and among the whole hypertensive patients group (including those who were not treated).* Diabetes* was defined as having fasting capillary blood glucose ≥ 7.0 mmol/l on 2 different days or the use of insulin or oral hypoglycemic drugs at the time of our survey and prediabetes was defined from 6.1 to 6.9 mmol/l [19, 20].

### 2.4. Ethical Approval

The research proposal was approved by Hanoi Medical University and three local district health departments (Phu Loc, Nam Dong, and Hue City in Thua Thien Hue Province). The study was explained to all respondents willing to participate and all participants gave their consent before participating in the study. All participants had the right to withdraw from the study at any time. Patients with new diagnostics of hypertension and/or diabetes were advised to consult their community health center or district hospital for the follow-up.

### 2.5. Statistical Analysis

Statistical analyses were performed to estimate the prevalence of hypertension and the associated risk factors. All statistical results were based on two-sided tests and *p* value < 0.05 was considered to represent statistical significance. To compare the difference between continuous variables, a *t*-test for two independent samples was used, and for categorical variables a chi-square test was used. Multivariate logistic regression analysis was performed to examine predictors of prevalence, awareness, treatment, and control of hypertension. The independent variable entry included gender (man, woman), age group (40–49, 50–59, and 60–69), marriage (married, unmarried, and widow(er)), residence (urban versus rural), ethnicity (Kinh majority versus minority), occupation (manual work, governmental staff, other, or no occupation), educational level (primary school or under, secondary or high school, and college or university), current smoking (yes versus no), physical inactivity (yes versus no), excessive alcohol consumption (yes versus no), BMI (overweight or obesity, yes versus no), abdominal obesity (yes versus no), and diabetes (yes versus no). A stepwise conditional logistic regression analysis was performed in order to select variable adopted in the multivariate regression model. EpiData entry (version 3.1, EpiData Association, Denmark) was used to record the study data. Descriptive, analytical, and multivariate logistic regression statistical analyses were carried out using IBM SPSS (version 23.0; IBM Corp., Armonk, NY, USA).

## 3. Results

### 3.1. Population Characteristics

Of the 1200 invited people, 983 participated, resulting in a 81.9% response rate. After excluding 14 records with missing information on hypertension risk factors, 969 participants were included for analysis. They had a mean age of 55.5 years (standard deviation: 8.8); 43,9% were men (Table 1). Most participants were married (87.0%); most had an educational background of primary school or under (59,6%) and were manual workers (46.0%). The mean SBP and DBP were 129.6 and 81.9 mmHg, respectively; SBP increased with age in both genders; DBP increased from 40–49 years to 50–59 years but decreased again in the oldest age group. Blood pressure levels of men were significantly higher than those of women (*p* < 0.001).

The prevalence of current smoking and that of excessive alcohol consumption were 33% and 7.6% in the corresponding order. These proportions in men were significantly higher than in women (Table 1). Notwithstanding, the proportions of physical inactivity were similar in both genders. More than half of the participants had excessive abdominal fat and the proportion of overweight, including obesity, was one-third of the participants. These proportions were higher in women than in men. The prevalence of diabetes accounted for 5.0%; there was no significant difference between both genders.

As regards health-seeking behavior, the hypertensive patients mainly attended the local community health centers (61.3%) and much less a private clinic (15.1%), district hospital (11.3%), provincial hospital (5.3), or private pharmacy (6.5%).

### 3.2. Prevalence, Awareness, Treatment, and Control of Hypertension

Table 2 provides the prevalence of hypertension according to gender, age group, place of residence, and other social determinants. Overall, the prevalence of hypertension in the age group of 40–69 years in the Vietnamese population was 44.8%. It was significantly higher in men than in women (51.3% versus 39.7%,* p *< 0.001). Hypertension's prevalence progressively increased with age. There was no significant difference in the prevalence of hypertension between rural and urban residents or according to educational level. However, hypertension prevalence was higher in the ethnic minority groups than among those from the ethnic majority (52.6% versus 41.5%,* p* < 0.001).

Overall, two-thirds of the participants with hypertension were aware of their hypertension. This proportion increased with age and ranged from 37.3% at age of 40 to 49 years to 78.3% at age of 60 to 69 years. The proportion of hypertension treatment was less than 50% among participants who were aware of their hypertension and one-third among all participants with hypertension. These proportions were higher in women than in men (see Table 2). Among those who were aware of their hypertensive condition and were accordingly well treated, only one-third achieved control of hypertension. The proportion of treated women was significantly higher than that of treated men (44.4% versus 27.0%,* p* = 0.03). Among all participants with hypertension, the prevalence of controlled hypertension achieved 12.2% and was significantly higher in women than in men (16.7% versus 7.8%,* p* < 0.001). The proportion of participants with controlled hypertension also increased with age; however, there was no significant difference among the age groups.

The prevalence and awareness of hypertension are similar among participants from urban and rural areas. On the contrary, the proportions of treatment and control of hypertension were significantly higher in urban areas than in rural areas (46.4% versus 26.1% and 21.2% versus 7.4%, resp.). The prevalence of hypertension in ethnic minorities was higher than that in the Kinh majority of the population. On the contrary, the treatment and control of hypertension were not lower in ethnic minorities than in the majority. There were no significantly different prevalence, awareness, treatment, and control of hypertension according to the educational level.

The prevalence of hypertension in people with physical inactivity, overweight and obesity, abdominal obesity, and diabetes was higher than that in people without those risk factors. There was no significant difference in terms of the awareness, treatment, and control of hypertension between the groups with and without physical inactivity, overweight and obesity, abdominal obesity, or diabetes, respectively. On the other hand, the prevalence of hypertension was not higher in people who were currently smoking or admitted excessive alcohol consumption. The control of hypertension among current smokers or excessive alcohol users was significantly lower than that in the group without these characteristics (Table 2).

### 3.3. Prevalence, Awareness, Treatment, and Control of Hypertension and Associated Risk Factors

The results from the multivariate logistic regression analysis (Table 3) showed that gender, age, ethnicity, BMI, abdominal obesity, and diabetes were independently associated with hypertension. Gender, age, and excessive alcohol consumption were independently associated with awareness of hypertension. Ethnicity, age, and residence were independently associated with treatment among patients who were aware of their hypertension. Age, residence, and excessive alcohol consumption were independently associated with treatment among all hypertensive patients. Residence was independently associated with control among patients with treated hypertension. Gender, age, and residence were independently associated with control of hypertension among all hypertensive patients.

## 4. Discussion

### 4.1. Sociodemographic Characteristics and Hypertension

This study found a large gap between the prevalence, the awareness, the treatment, and the adequate control of hypertension in (Central) Vietnam. Nearly half of individuals were diagnosed with hypertension. However, more than one-third of the hypertensive patients did not know their blood pressure condition; only half of the aware (meaning one-third of all hypertensive patients) had received treatment. No more than one-third of the treated hypertensive (meaning one in eighth of all hypertensive patients) had been adequately controlled. This pattern was consistent with the results of national surveys previously reported in Vietnam and other low- and middle-income countries [3, 14, 21]. For example, the awareness, treatment, and control of hypertension in the low- and middle-income countries were 43,6%, 36,9%, and 9,9%, respectively, according to the PURE study [21]. Previously, poor access to medical facilities combined with limited availability of adequate treatment was shown to be correlated with hypertension in Vietnam and Southeast-Asian population. In addition to the population's lack of knowledge about hypertension, the relationship between physicians and patients, the adherence to medication, and health system characteristics were suggested to play an important role in the attitude of the population to the disease [22–24].

The prevalence of hypertension in our study was higher than that in previous studies in Vietnam (44.8% versus 25.1%). This could be explained by the previous studies sampled in a population aged 25 years or above and in different regions in Vietnam. During the last decades, there has been increasing prevalence of hypertension among Vietnamese citizens [3, 7, 13, 14]. However, reassuringly, the prevalence of hypertension in this study was rather similar to age-standardized prevalence reported in cross-sectional national study [3]. The proportion of awareness with age-standardization in our study was higher than those in the other studies in Vietnam [3, 14]. Between those two sets of studies, there have been a lot of activities to raise the local people's awareness of hypertension. Health education campaigns have appeared on national as well as local television channels and in the newspapers [25, 26]. As a consequence, the population was sensitized about hypertension and hypertensive patients could be detected earlier. However, our proportion of treatment and control of hypertension was similar to the previous study in Vietnam and in other low- middle-income countries. There was no increase in the proportion of treatment or the control of hypertension either. These findings were far from optimal, a disturbing trend that has been observed in many other developing countries; for example, the proportion of awareness, treatment, and control in Canada in 2013 were 84.3%, 80%, and 68%, respectively [7, 8, 21]. Hypertension management is still a big challenge in Central Vietnam. In order to promote hypertension treatment and control, it is desirable to make people more aware of the risks of hypertension. Further, research should explore the barriers of optimal management of hypertension.

The overall mean SBP and DBP were significantly higher in men than in women and rose progressively with age in both genders. The prevalence of hypertension was also significantly higher in men than in women. However, the proportions of awareness, treatment, and control were higher in women than in men. Of particular concern is the proportion of awareness, treatment, and control of hypertension in young adults aged 40 to 49 years, which was substantially lower than in older adults. The early detection of hypertension in young adults should be conducted more regularly. This was consistent with other studies in Vietnam and in other low-, middle-, and high-income countries [3, 7, 11, 21]. In the multivariate logistic regression analysis, gender and age were independently associated with prevalence, awareness, and control of hypertension. Only age and not gender was found to be independently associated with the treatment of hypertension.

The prevalence and awareness of hypertension in urban and rural areas appeared to be similar (45.8% versus 44.3% and 68.2% versus 66.0%). These results were not consistent with the previous findings in Vietnam with much higher proportions in urban areas than in rural areas (32.7% versus 17.3% and 58.4% versus 28.8%) [3]. This situation might be related to economic development in rural areas, and associated lifestyle and diet changes lead to an increased prevalence and awareness of hypertension. However, the treatment and control were much higher in urban areas than in rural areas, and place of residence was an independent risk factor for treatment and control of hypertension. These findings were consistent with the results of the low- and middle-income countries such as China, Colombia, and Iran but were different from those of the high-income countries like Canada or Sweden [21]. In many developing countries, the treatment and control of hypertension were not only a burden for the health service in the rural areas, despite that their prevalence still remained lower than that in the urban areas [21]. In Vietnam, the prevalence of hypertension in rural areas has increased from 14.1% in 2005 to 22% in 2011, but the health services have not yet sufficiently developed to meet the population's needs [12, 13]. It was also suggested that the socioeconomic status gap had a strong influence on hypertension management. It requires improving the healthcare provision in rural areas to cope with the new challenges. Among the ethnic minority groups, there was high prevalence of hypertension, but the control and treatment were largely insufficient. Most of the ethnic minorities are living in the mountainous and remote rural areas, facing difficulties such as low income, poor health education, insufficient health facilities, difficult access to medical services, and lack of health resources [24, 27, 28]. There was no association between the level of education and prevalence, awareness, treatment, and control of hypertension in this study.

It was shown in our study that the community health centers were most frequently visited by hypertensive patients (61.3%) and that the other types of health facilities were much less popular. This does not necessarily indicate the differences in the quality of care; rather, it might have been because of the easier access to the community health centers and the fact that these centers were mainly in charge of providing primary healthcare, particularly hypertension treatment. Under any circumstances, the community health centers should therefore be considered an important stakeholder to increase the proportion of treatment and control of hypertension.

### 4.2. Health Behavior and Metabolic Risk Factors and Hypertension

One of the factors that could contribute to the high prevalence of hypertension in Vietnam was the increasing prevalence of overweight and obesity. The prevalence of overweight and obesity in adults increased from 2005 (standing at 19.8% in men and 21.9% in women) to 2008 (accounting for 18.4% in men and 22.7% in women) [3, 14]. Overweight including obesity and abdominal obesity has become highly prevalent in developed countries and has been rapidly reaching epidemic proportions in the developing ones. The high prevalence in our study, which was higher than the results of the previous research in Vietnam, could be related to the fact that the mean age in our sample was higher. The waist circumference is increasing with age in adults of both sexes up to the age of 70 years. The age-related differences in waist hip ratio were also reported in all BMI categories examined in both men and women. The increasing rate of obesity might as well be attributed to the fast urbanization, industrialization, and modernization [17, 18]. Also described in the present study was higher prevalence of hypertension in the overweight and obesity group as compared to those without overweight and obesity. In the multivariate logistic regression analysis, there was an independent association between obesity or abdominal obesity and hypertension. This finding was consistent with previous literature [9, 14, 29].

In this study, hypertension was more commonly diagnosed in the patients with diabetes type 2 as compared to the general population. High blood pressure was detected among over two-thirds of the patients with diabetes type 2, and its development coincided with the development of hyperglycemia. Diabetes was notified to be independently associated with hypertension like in previous studies [9, 30]. Simultaneously, a higher rate of hypertension was detected in the group with physical inactivity. As physical activity had a positive effect on many risk factors such as hypertension, obesity, and diabetes, regular physical exercise would reduce the risk of many adverse health outcomes (all-cause and CVD mortality) over a wide range of age [9]. Therefore, in order to reduce the risk of obesity, hypertension, and diabetes type 2, there should be a promotion of physical activities in the community.

In addition, according to our study, one-third of the study population, mostly men, had been currently smoking. The prevalence of hypertension in current smokers was not significantly higher than that in those without current smoking. There has been little if any association between the current smoking activity and the prevalence, awareness, treatment, and control of hypertension in the multivariate logistic regression analysis. However, among the treated hypertension patients, blood pressure was better controlled among noncurrent smokers than among current smokers. Therefore, the antismoking campaigns by the Ministry of Health, the Vietnamese Government, and the National Assembly's Policies should continue [24, 31]. In our study, men showed high prevalence of excessive alcohol consumption. People without excessive alcohol use were more likely to be aware of the risks related to hypertension. According the multivariate logistic regression analysis, excessive alcohol consumption was independently associated with diagnosed hypertension and treatment among all hypertensive patients. Previous studies also indicated that hypertension was higher in those with excessive alcohol consumption [13]. Hypertension awareness, treatment, and control should be integrated in the strategy of hypertension management for alcohol consumers.

### 4.3. Limitations and Strengths

According to the definition, the diagnosis of hypertension should have been based on two or more blood pressure readings taken at two or more visits after the initial screening [18]. In our study, blood pressure was measured in a single visit, so the prevalence of hypertension might have been overestimated and the control of hypertension underestimated. However, this common problem of large epidemiological study might have some effects on within sample comparisons, especially when a standard protocol and analysis were applied. The information collected through the interview may be inaccurate due to the education level and recall bias of the participants. Effective quality control procedures of blood pressure measurement and the interview were held by well-trained investigators to reduce the possibility of attrition bias.

In our study, there was a slight chance of misclassification of diabetes status, since it was determined by self-report or two hyperglycemic readings with a capillary glucometer. Fortunately, it was likely to be acceptable in such a setting with limited resources like ours [25, 26]. A comprehensive picture of hypertension and its risk factors among the Vietnamese population aged 40 and over in this research was then guaranteed.

## 5. Implication of Study

This study has several important implications for public health staff, clinicians, and health policy makers. It was shown from our findings that hypertension still remained a serious public health problem in Central Vietnam with high prevalence of hypertension and low level of awareness, treatment, and control. This result contributes to the ongoing policy debate with respect to the prevention and control of hypertension in Vietnam. In particular, it ascertained the necessity to improve the effectiveness of the current National Targeted Program for Prevention and Control of Hypertension. This program should focus more on the hypertensive patients in rural areas and the ethnical hypertensive groups because they showed high prevalence of hypertension and low proportion of treatment and control. This study also showed high prevalence of overweight and obesity, current smoking, and alcohol consumption. Therefore, clinicians should consider both nonmedication and medication approaches for treatment of hypertension. And such efforts might as well include the use of mass media to educate the general population, delivering brochures and leaflets to hypertensive patients and providing leadership from social and professional organizations to promote healthy lifestyles. Improving the capacity of the community health centers would be an important activity for enhancing the rate of early detection, treatment, and control of hypertension. Further studies could be conducted to explore the barriers of hypertension management from the healthcare provider and patient's perspective as well as the new community-based intervention approaches to find out the possible solutions to the present situation in (Central) Vietnam.

## 6. Conclusion

To summarize, the findings of this study indicated that hypertension remained an important public health problem in Central Vietnam. In spite of the increasing prevalence of hypertension over the past 10 years in (Central) Vietnam, the treatment and effective control of hypertension had remained unacceptably low. Nearly half of the adult Vietnamese population aged from 40 to 69 years was hypertensive, but only one-third of the hypertensive patients received due treatment, and not more than one in eighth of all hypertensive patients had been adequately controlled. Age, gender, place of residence, body mass index, and diabetes were found to be independent risk factors for hypertension. These findings suggested that early detection, raising awareness, and providing better treatment of hypertension could be very important to maintain and improve the quality of hypertension management, especially in rural areas to reduce the burden of hypertension. Moreover, the lifestyle modifications including the prevention of overweight and obesity, smoking, and alcohol consumption could help prevent hypertension.

## Figures and Tables

**Table 1:** Characteristics of the study population aged 40–69 years by gender.

Characteristics [mean (SD) or *n* (%)]	Men	Women	Total
*Age (in years)*	56.2 (8.9)	55.0 (8.7)	55.5 (8.8)
*BMI (kg/m* ^*2*^)	21.6 (3.0)	22.3 (3.2)	22.0 (3.1)
*Hip waist ratio*	0.88 (0.06)	0.89 (0.07)	0.89 (0.07)
*Blood pressure*			
Systolic (mmHg)	135.0 (22.8)	125.3 (20.6)	129.6 (22.0)
40–49	128.6 (19.5)	118.1 (17.3)	122.7 (19.0)
50–59	134.5 (23.1)	125.3 (22.1)	128.8 (22.9)
60–69	139.2 (23.5)	131.1 (19.5)	135.2 (21.9)
Diastolic (mmHg)	85.6 (13.9)	79.0 (12.3)	81.9 (13.4)
40–49	84.5 (14.8)	77.1 (10.6)	80.3 (13.1)
50–59	87.4 (14.5)	80.5 (14.0)	83.1 (14.5)
60–69	85.1 (12.9)	78.8 (11.1)	82 (12.4)
*Age groups*			
40–49	111 (26.1)	146 (26.8)	257 (26.5)
50–59	131 (30.8)	215 (39.5)	346 (35.7)
60–69	183 (43.1)	183 (33.6)	366 (37.8)
*Marital status *			
Married	408 (96.0)	435 (80.0)	843 (87.0)
Unmarried, Widow(er)	17 (4.0)	109 (20.0)	126 (13.0)
*Residence*			
Urban	139 (32.7)	191 (35.1)	330 (34.1)
Rural	286 (67.3)	353 (64.9)	639 (65.9)
*Ethnicity*			
Ethnic majority (Kinh)	301 (70.8)	385 (70.8)	686 (70.8)
Ethnic minorities	124 (29.2)	159 (29.2)	283 (29.2)
*Educational level*			
Primary school or under	193 (45.4)	385 (70.8)	578 (59.6)
Middle or high school	201 (47.3)	141 (25.9)	342 (35.3)
College or university	31 (7.3)	18 (3.3)	49 (5.1)
*Occupation*			
Manual workers	217 (51.1)	217 (39.9)	434 (44.8)
Government staff	107 (25.2)	55 (10.1)	162 (16.7)
Other occupations	101 (23.8)	272 (50.0)	373 (38.5)
*Current smoking status*			
*Yes*	225 (52.9)	95 (17.5)	320 (33.0)
*No*	200 (57.1)	449 (82.5)	649 (67.0)
*Excessive alcohol consumption*			
*Yes*	69 (16.2)	5 (0.9)	74 (7.6)
*No*	356 (83.8)	539 (99.1)	895 (92.4)
*Physical activity level*			
*Low*	44 (10.4)	64 (11.8)	108 (11.1)
*Moderate*	73 (17.2)	85 (15.6)	158 (16.3)
*High*	308 (72.5)	398 (72.6)	703 (72.6)
*Body mass index*			
*Underweight*	68 (16.0)	56 (10.3)	124 (12.7)
*Normal weight*	219 (51.5)	272 (50.0)	491 (50.7)
*Overweight, obesity *	138 (32.5)	216 (39.7)	354 (36.5)
*Abdominal obesity*			
*Yes*	164 (38.6)	384 (70.6)	548 (56.6)
*No*	261 (61.4)	160 (29.4)	421 (43.3)
*Blood glucose*			
*Diabetes*	23 (5.4)	25 (4.6)	48 (5.0)
*Prediabetes*	43 (10.1)	39 (7.2)	82 (8.5)
*No*	359 (84,5)	480 (88.2)	839 (86.5)

BMI: body mass index, SD: standard deviation.

**Table 2:** Prevalence, awareness, treatment, and control of hypertension of the adult population aged 40–69 years.

Characteristics	Number of participants (*N*)	Prevalence of hypertension *N*1 (*A*%) (*A* = *N*1/*N*)	Awareness of hypertension *N*2 (*B*%) (*B* = *N*2/*N*1)	Treated hypertension		Controlled hypertension	
				Among awareness *N*3 (*C*%) (*C* = *N*3/*N*2)	Among All hypertensive *N*3 (*D*%) (*D* = *N*3/*N*1)	Among treated *N*4 (*E*%) (*E* = *N*4/*N*3)	Among All hypertensive *N*4 (*F*%) (*F* = *N*4/*N*1)
**Total**	969	434 (44.8)	292 (67.3)	144 (49.3)	144 (33.2)	53 (36.8)	53 (12.2)
*Gender (p value) *		<0.001	0.001	0.2	0.01	0.03	<0.001
Men	425	218 (51.3)	131 (60.1)	63 (48.1)	63 (28.9)	17 (27.0)	17 (7.8)
40–49	111	41 (36.9)	11 (26.8)	1 (9.1)	1 (2.4)	0	0
50–59	131	66 (50.4)	39 (59.1)	19 (48.7)	19 (28.8)	4 (21.1)	4 (6.0)
60–69	183	111 (60.7)	81 (73.0)	43 (53.1)	43 (38.7)	13 (30.2)	13 (11.7)
Women	544	216 (39.7)	161 (74.5)	81 (50.3)	81 (37.5)	36 (44.4)	36 (16.7)
40–49	146	33 (22.6)	17 (51.5)	7 (41.2)	7 (21.2)	3 (42.9)	3 (9.1)
50–59	215	91 (42.3)	66 (72.5)	29 (43.9)	29 (31.9)	14 (48.3)	14 (15.4)
60–69	183	92 (50.3)	78 (84.8)	45 (57.7)	45 (48.9)	19 (42.2)	19 (20.6)
*Age group*		<0.001	<0.001	0.06	<0.001	0.86	0.42
40–49	257	74 (28.8)	28 (37.3)	8 (28.6)	8 (10.8)	4 (44.4)	4 (5.3)
50–59	346	157 (45.4)	105 (66.9)	48 (45.7)	48 (30.6)	18 (37.5)	18 (11.5)
60–69	366	203 (55.5)	159 (78.3)	88 (55.3)	88 (43.3)	31 (35.6)	31 (15.3)
*Ethnicity*		0.001	0.3	<0.001	<0.001	0.06	0.001
Ethnic majority	686	285 (41.5)	187 (65.6)	110 (58.8)	110 (38.6)	45 (40.9)	45 (15.8)
Ethnic minority	283	149 (52.6)	105 (70.5)	34 (32.4)	34 (22.8)	8 (23.5)	8 (5.4)
*Marital status*		0.9	0.004	0.33	0.64	0.25	0.54
Married	843	378 (44.8)	245 (64.8)	123 (50.2)	123 (32.5)	47 (38.2)	47 (12.4)
Unmarried, Widow(er)	126	56 (44.4)	47 (83.9)	20 (42.6)	20 (35.7)	5 (25.0)	5 (8.9)
*Residence*		0.6	0.7	<0.001	<0.001	0.03	<0.001
Urban	330	151 (45.8)	103 (68.2)	70 (68.0)	70 (46.4)	32 (45.7)	32 (21.2)
Rural	639	283 (44.3)	189 (66.8)	74 (39.2)	74 (26.1)	21 (28.4)	21 (7.4)
*Educational level *		0.32	0.16	0.49	0.29	0.44	0.68
Primary school or under	578	254 (43.9)	180 (70.9)	84 (46.7)	84 (33.1)	33 (39.3)	33 (13.0)
Middle or high school	342	153 (44.7)	96 (62.7)	52 (54.2)	52 (34.0)	16 (30.8)	16 (10.5)
College or university	49	27 (55.1)	16 (59.3)	8 (50.0)	8 (47.1)	4 (50.0)	4 (14.8)
*Occupation*		<0.001	0.3	<0.001	<0.001	0.33	0.004
Manual worker	434	177 (39.4)	114 (64.3)	39 (35.4)	39 (22.8)	12 (28.2)	12 (6.4)
Government staff	162	98 (59.3)	64 (64.6)	36 (53.2)	36 (34.4)	12 (33.3)	12 (11.5)
Other occupations	348	159 (45.7)	114 (71.7)	69 (60.5)	69 (43.4)	29 (42.0)	29 (18.2)
*Current smoking*		0.7	0.5	0.39	0.65	0.03	0.04
Yes	320	146 (45.6)	101 (69.2)	46 (45.5)	46 (31.5)	11 (23.9)	11 (7.5)
No	649	288 (44.4)	191 (66.3)	98 (50.8)	98 (33.7)	42 (42.3)	42 (14.2)
*Excessive alcohol consumption*		0.154	<0.001	0.31	0.005		0.016
Yes	74	39 (52.7)	14 (5.9)	5 (35.7)	5 (12.8)	0	0
No	895	395 (44.1)	278 (70.4)	138 (49.6)	138 (34.9)	52 (37.7)	52 (13.2)
*Physical inactivity*		0.029	0.32	0.5	0.89	0.57	0.69
Yes	108	59 (54.6)	43 (72.9)	19 (44.2)	19 (32.2)	8 (42.1)	8 (13.6)
No	861	375 (43.6)	249 (66.4)	124 (49.8)	124 (33.1)	44 (35.5)	44 (17.2)
*Overweight, obesity*		<0.001	0.47	0.37	0.67	0.3	0.52
Yes	354	185 (52.3)	121 (65.4)	63 (52.1)	63 (34.1)	20 (31.7)	20 (10.8)
No	615	249 (40.5)	171 (68.7)	80 (46.8)	80 (32.1)	32 (40.0)	32 (12.9)
*Abdominal obesity*		0.011	0.068		0.025	0.44	0.5
Yes	548	265 (48.4)	187 (70.6)	98 (52.4)	98 (37.0)	34 (34.7)	18 (10.7)
No	421	169 (40.1)	105 (62.1)	45 (42.9)	45 (26.6)	18 (40.0)	34 (12.8)
*Diabetes*		0.001	0.025	0.28	0.96	0.19	0.25
Yes	48	33 (68.8)	28 (84.8)	11 (39.3)	11 (33.3)	6 (54.4)	6 (18.2)
No	921	401 (43.5)	264 (65.8)	132 (50.0)	132 (32.9)	46 (34.8)	46 (11.5)

**Table 3:** Multivariate logistic regression analysis demonstrating the relationship between the prevalence, awareness, treatment, and control of hypertension with several independent variables.

Risk factors	OR (95% CI)	*p* value
*Prevalence of hypertension*		
Gender (men versus women)	1.83 (1.37–2.44)	<0.001
Ethnicity (Kinh versus minority)	0.54 (0.40–0.73)	<0.001
Age group (per 10 years)	0.56 (0.47–0.67)	<0.001
BMI (overweight and obesity versus no)	1.82 (1.35–2.45)	<0.001
Abdominal obesity (yes versus no)	1.40 (1.03–1.91)	0.032
Diabetes (yes versus no)	2.18 (1.13–4.19)	0.019
*Awareness of hypertension*		
Gender (men versus women)	0.47 (0.29–0.77)	0.003
Age group (per 10 years)	0.42 (0.31–0.57)	<0.001
Excessive alcohol consumption (yes versus no)	0.33 (0.16–0.72)	0.005
*Treatment among aware hypertensive patients*		
Ethnicity (Kinh versus minority)	1.72 (0.95–3.12)	0.073
Age group (per 10 years)	0.62 (0.42–0.9)	0.013
Residence (urban versus rural)	2.6 (1.43–4.73)	0.002
*Treatment among all hypertensive patients*		
Age group (per 10 years)	0.47 (0.34–0.65)	<0.001
Residence (urban versus rural)	2.26 (1.43–3.58)	<0.001
Excessive alcohol consumption (yes versus no)	0.34 (0.13–7.93)	0.034
*Control among treated hypertension*		
Residence (urban versus rural)	2.14 (1.05–4.34)	0.035
*Control among all hypertensive patients*		
Gender (men versus women)	0.41 (0.21–0.79)	0.008
Age group (per 10 years)	0.49 (0.28–0.81)	0.005
Residence (urban versus rural)	3.49 (1.88–6.47)	<0.001

CI: confidence interval, BMI: body mass index.

## Data Availability

The data set is owned by Hue University of Medicine and Pharmacy and the research partners. The data set underlying the findings of the study is available on request from Nguyen Minh Tam at nmtam@huefmc.com or the corresponding author of this study.
